# P-1902. Routine Anti-Nuclear Antibody (ANA) Testing in Patients Experiencing Long COVID: Worth it?

**DOI:** 10.1093/ofid/ofae631.2063

**Published:** 2025-01-29

**Authors:** Abigail Ghorbani, Nora Strong, MaryJane Keller, Luis Ostrosky-Zeichner

**Affiliations:** McGovern Medical School at UTHealth Houston, Houston, Texas; McGovern Medical School at UTHealth Houston, Houston, Texas; McGovern Medical School at UTHealth Houston, Houston, Texas; McGovern Medical School. UTHealth, Texas, Texas

## Abstract

**Background:**

Patients with long COVID are often tested for ANA as part of their routine work-up (Son et al., 2023). However, it is unclear if this testing leads to a rheumatological diagnosis or is actionable. This project assessed the frequency of ANA-positivity in patients experiencing long COVID and the outcomes of rheumatic disease diagnosis and treatment.

**Methods:**

Electronic medical records (EMR) from January 2022 through May 2023 at the UT Physicians COVID Center of Excellence were reviewed. The population included those experiencing long COVID and presenting with symptoms. Symptoms, personal and family history of rheumatic disease, last known COVID date, ANA test results, referral to rheumatology, diagnoses, and treatment, if any, were recorded. Data was analyzed using Microsoft Excel PivotTables and JavaStat, with right-tailed Fisher Exact Tests.

**Results:**

The 117-patient sample was 76.92% female, with an average age of 49.21+/-15.09 SD years, with last known COVID between March 2020 and February 2023. 17.95% had a previous rheumatic diagnosis, and 22.22% had a family history of rheumatic disease. The symptoms reported were: fever (6.84%), arthralgia (43.59%), arthritis (22.22%), rash (23.08%), and fatigue (79.49%). 75.21% of patients received an ANA test, of which 43.18% were positive. The mode titer was 1:80, and the most common pattern was nuclear. 48.72% of the patients with a positive test were referred to rheumatology, but only 21.05% received a rheumatological diagnosis. Only 11.97% received any treatment for their symptoms, and most treatments were for symptom control or an anti-inflammatory. On the last visit, only 2.56% had resolution of long COVID symptoms. None of the symptoms were predictive of a positive ANA test.

**Conclusion:**

The majority of patients experiencing long COVID presenting to our clinic received an ANA test, and almost half had a positive result. Few received any rheumatological diagnosis and most had ongoing symptoms by their latest visit. Although this study is limited by sample size, bias in ANA ordering, and its retrospective nature, these data suggest that routine ANA testing and rheumatology referrals may not be cost-effective in patients experiencing long COVID.

**Disclosures:**

Luis Ostrosky-Zeichner, MD, FACP, FIDSA, FSHEA, FECMM, CMQ, Cidara: Advisor/Consultant|Enanta: Advisor/Consultant|F2G: Advisor/Consultant|Gilead: Advisor/Consultant|GSK: Advisor/Consultant|Melinta: Advisor/Consultant|Octapharma: Advisor/Consultant|Pfizer: Advisor/Consultant|Pfizer: Grant/Research Support|Pulmocide: Grant/Research Support|Scynexis: Grant/Research Support|Viracor: Advisor/Consultant

